# Evidence of *Mycobacterium bovis* DNA in shared water sources at livestock–wildlife–human interfaces in KwaZulu-Natal, South Africa

**DOI:** 10.3389/fvets.2025.1483162

**Published:** 2025-02-28

**Authors:** Megan C. Matthews, Deborah M. Cooke, Tanya J. Kerr, Andre G. Loxton, Robin M. Warren, Giovanni Ghielmetti, Elizabeth M. Streicher, Carmel S. Witte, Michele A. Miller, Wynand J. Goosen

**Affiliations:** ^1^South African Medical Research Council Centre for Tuberculosis Research, Division of Molecular Biology and Human Genetics, Faculty of Medicine and Health Sciences, Stellenbosch University, Cape Town, South Africa; ^2^Section of Veterinary Bacteriology, Institute for Food Safety and Hygiene, Vetsuisse Faculty, University of Zurich, Winterthurerstrasse, Zürich, Switzerland; ^3^Department of Microbiology and Biochemistry, Faculty of Natural and Agricultural Sciences, University of the Free State, Bloemfontein, South Africa

**Keywords:** culture-independent detection, environmental transmission, *Mycobacterium bovis*, *Mycobacterium tuberculosis* complex, targeted next generation sequencing, One Health, Oxford Nanopore Technologies

## Abstract

The *Mycobacterium tuberculosis* complex (MTBC) including *Mycobacterium bovis* (*M. bovis*), which primarily affects animal hosts; however, it is also capable of causing zoonotic infections in humans. Direct contact with infected animals or their products is the primary mode of transmission. However, recent research suggests that *M. bovis* can be shed into the environment, potentially playing an under-recognized role in the pathogen’ spread. Further investigation into indirect transmission of *M. bovis*, employing a One Health approach, is necessary to evaluate its epidemiological significance. However, current methods are not optimized for identifying *M. bovis* in complex environmental samples. Nevertheless, in a recent study, a combination of molecular techniques, including next-generation sequencing (NGS), was able to detect *M. bovis* DNA in the environment to investigate epidemiological questions. The aim of this study was, therefore, to apply a combination of culture-independent methods, such as targeted NGS (tNGS), to detect pathogenic mycobacteria, including *M. bovis*, in water sources located in a rural area of KwaZulu-Natal (KZN), South Africa. This area was selected based on the high burden of MTBC in human and animal populations. Water samples from 63 sites were screened for MTBC DNA by extracting DNA and performing *hsp65* PCR amplification, followed by Sanger amplicon sequencing (SAS). Sequences were compared to the National Centre for Biotechnology Information (NCBI) database for genus or species-level identification. Samples confirmed to contain mycobacterial DNA underwent multiple PCRs (*hsp65*, *rpoB*, and MAC *hsp65*) and sequencing with Oxford Nanopore Technologies (ONT) tNGS. The ONT tNGS consensus sequences were compared to a curated in-house database to identify mycobacteria to genus, species, or species complex (e.g., MTBC) level for each sample site. Additional screening for MTBC DNA was performed using the GeneXpert® MTB/RIF Ultra (GXU) qPCR assay. Based on GXU, *hsp65* SAS, and ONT tNGS results, MTBC DNA was present in 12 of the 63 sites. The presence of *M. bovis* DNA was confirmed at 4 of the 12 sites using downstream polymerase chain reaction (PCR)-based methods. However, further studies are required to determine if environmental *M. bovis* is viable. These results support further investigation into the role that shared water sources may play in TB epidemiology.

## 1 Introduction

The *Mycobacterium tuberculosis* complex (MTBC) includes *M. tuberculosis* (MTB) and *Mycobacterium bovis* (*M. bovis*), which primarily infect human and animal hosts, respectively (1). In addition, MTB can spread from humans to both domestic and wild animals, resulting in reverse zoonosis (2, 3). Similarly, *M. bovis* can lead to zoonotic tuberculosis (ZTB) infections, whih account for an estimated 12% of human TB cases globally (4). In African countries and among pastoralist communities where unpasteurized milk is consumed, the rate of ZTB can be higher, up to 37.7% of cases (5).

The spread of *M. bovis* occurs primarily by prolonged close contact with infected hosts through aerosols (6). However, shed bacilli may persist in environmental material for 1–3 months (7) and contribute to indirect transmission (8). Indirect transmission of *M. bovis* between animal hosts through shared food sources has been demonstrated experimentally (9). Furthermore, the presence of *M. bovis* in invertebrates, soil, and shared water sources near infected host populations suggests that environmental transmission may occur (8, 10–12). Evaluating epidemiological links for TB between people, animals, and their shared environment requires a One Health approach (13). However, detecting *M. bovis* in the environment poses a challenge due to the complexity and paucibacillary nature of the samples. In addition, the survival of *M. bovis* is influenced by variable environmental conditions (14, 15).

Other mycobacterial species also play a vital role in human and animal health. Non-tuberculous mycobacteria (NTMs) are ubiquitous in the environment but can lead to opportunistic infections in humans and animals (2). Infections caused by NTMs have been increasingly reported, especially in immunocompromised individuals (16–18). Members of the *Mycobacterium avium* complex (MAC) are common NTMs that have been implicated in human and animal diseases. For example, the MAC subspecies paratuberculosis causes Johne’s disease in livestock and has been associated with Crohn’s disease in humans (19, 20). Furthermore, exposure of human and animal populations to NTMs can result in cross-reactive immune sensitization, which may impact MTBC vaccine responses and the accuracy of diagnostic tests (21, 22).

Infections caused by different mycobacterial species (e.g., MTBC or NTMs) or ecotypes (e.g., MTB or *M. bovis*) may differ in pathogenicity, host species immune responses, and antibiotic resistance, requiring different diagnostic and management approaches. Therefore, the correct identification of mycobacterial species and ecotype is, crucial for favorable treatment outcomes (23, 24). Antemortem detection of MTBC infection currently depends on cytokine release assays, tuberculin skin tests, thoracic radiographs, and screening with the GeneXpert® MTB/RIF Ultra (GXU Cepheid, CA, USA) quantitative polymerase chain reaction (qPCR) assay (25, 26). However, these techniques cannot differentiate between infections caused by different MTBC ecotypes or strains (27, 28). Additionally, co-infections caused by NTMs may result in cross-reactive immunological responses, which can confound TB diagnostic test interpretation, especially in areas with high human and animal TB, as well as environmental NTM, burdens (29, 30). Therefore, culture and characterization of mycobacterial isolates remain the gold standard for detecting MTBC despite limitations such as the paucibacillary nature of antemortem samples, lengthy incubation times, and biosafety concerns associated with handling viable mycobacteria (31, 32).

Advances in molecular techniques, such as quantitative PCRs (qPCR) and PCR amplicon-targeted next-generation sequencing (tNGS), have improved the sensitivity of mycobacterial detection in complex samples (12, 33). In contrast to qPCR, amplicon tNGS is highly scalable and more suitable for characterizing multiple gene targets in a complex microbiome (34). Specific gene targets for genus-level detection of mycobacteria include DNA-directed RNA polymerase subunit beta (*rpoB*) and a heat shock protein of 65-kDa (*hsp65*) (35, 36). The variation within these gene targets can be used for species-level identification. Moreover, sequencing additional gene targets can be used to differentiate MAC and MTBC ecotypes, including MAC *hsp65* and gyrase (*gyrA*, *gyrB1*, and *gyrB2*) gene regions, respectively (37, 38). Oxford Nanopore Technologies (ONT, Oxford, UK) NGS has increased the resolution of phylogenetic comparisons of *M. bovis* isolates (33, 39) to elucidate epidemiological links in much the same way that spacer oligonucleotide typing (spoligotyping) have been used for cultured isolates historically (40).

Previous studies have detected NTMs and MTBC in human and animal populations at livestock-wildlife-human interfaces in KwaZulu-Natal, South Africa (41–44). There is a high burden of MTB in humans and *M. bovis* in animals in this area and spillovers into the environment could occur and play an under-recognized role in MTBC epidemiology. Therefore, this study aimed to determine if *M. bovis* DNA was present in shared water sources in a rural area of KwaZulu-Natal, South Africa. This is the first step in evaluating if *M. bovis* or MTB are being shed into this environment, using culture-independent methods.

## 2 Materials and methods

### 2.1 Location and sample collection

The study areas included rural communities that span two municipalities within the Umkhanyakude District, KwaZulu-Natal province, South Africa. Shared water sources, with observed animal and human usage, were sampled in the Mtubatuba municipality (*n* = 14) and Big Five Hlabisa municipality (*n* = 49). These included areas surrounding local schools, residences, community areas, medical facilities, and farming areas (Supplementary Figures S1, S2). The Mtubatuba municipality communities border the Hluhluwe-iMfolozi Park (HiP), which contains African buffalo (*Syncerus caffe*) populations that are endemically infected with *M. bovis* (45). Rural communities in these areas practice communal grazing, where *M. bovis*-infected cattle (*Bos taurus*) and goats (*Capra hircus*) have been identified (41, 46). One to eight environmental samples were collected from each site into 50 mL conical bottom centrifuge tubes (Abdos Life Sciences, Roorkee, Uttarakhand, India). These samples were collected from the water’s edge in a single scooping motion that included a mixture of sediment and water. Water sources were not filtered or treated with any wastewater treatment prior to sample collection. All samples were boiled at 98°C for 45 min, as required for transport of samples to Stellenbosch University, where they were stored at 4°C prior to downstream molecular testing.

### 2.2 Detection and characterization of mycobacterial DNA

#### 2.2.1 DNA extraction

Prior to DNA extraction, a 10 mL aliquot from each sample was centrifuged at 3,200 rcf for 10 min in an Eppendorf 5810R (Eppendorf, Hamburg, Germany) to concentrate any microorganisms present in the sample. The supernatant was decanted, and 0.25 g of the pellet was used for DNA extraction with the Qiagen DNeasy PowerSoil Pro kit (Qiagen, Hilden, Germany), according to the manufacturer’s guidelines. Cell lysis was performed for 20 min using a Vortex-Genie 2 (Scientific Industries, Bohemia, NY, USA) and a 12-tube adapter (Macherey-Nagel, Düren, Nordrhein-Westfalen, Germany). A final volume of 60 μL of genomic DNA was eluted per sample and stored at −20°C. The quantity (ng/μL) of extracted DNA was determined for 20 randomly selected samples using the Qubit DNA Broad Range Assay Kit and the Qubit™4 Fluorometer (both ThermoFisher Scientific, Waltham, MA, USA), according to the manufacturer’s instructions. Moreover, the integrity of the extracted DNA was evaluated by electrophoresis using a 1% agarose gel. Gel imaging was conducted using the BioRad Chemi Doc Universal Hood III and Gel Documentation System (Bio-Rad Laboratories, Hercules, CA, USA) and the BioRad Image Lab 6.1 Software.

#### 2.2.2 PCR amplification

A universal bacterial *16S* PCR assay was used to assess if DNA samples could be amplified and to confirm the absence of PCR inhibitors (47). Briefly, each PCR reaction consisted of 9.5 μL of nuclease-free water, 12.5 μL OneTaq Hot start 2× master mix with standard buffer (New England Biolabs, Ipswich, MA, USA), 0.5 μL forward primer, 0.5 μL reverse primer, and 2 μL of DNA template, in a total volume of 25 μL. Primers were ordered from Integrated DNA Technologies (IDT, IO, USA) and comprise a working stock concentration of 10 μL. Cycling conditions consisted of 1 cycle at 94°C for 10 min, followed by 35 cycles of denaturation at 94°C for 30 s, annealing at 65°C for 30 s, elongation at 68°C for 90 s, and a final elongation step of 5 min at 72°C, using an Applied Biosystems Veriti 96-well Fast Thermal Cycler (ThermoFisher Scientific Waltham, MA, USA). Gel electrophoresis was conducted to confirm that amplicons were the expected sizes. If DNA could be amplified using the *16S* PCR assay, PCR was repeated with primers targeting additional gene targets (*hsp65*, *rpoB*, MAC *hsp65*, and *gyrase*) with optimized cycling conditions (Table 1). All PCRs conducted in this study included a positive control (*M. bovis* DNA) and a non-template control (nuclease-free water). If DNA could not be amplified with *16S* PCR, it was not used in downstream molecular testing.

**Table 1:** Published polymerase chain reaction (PCR) primers for mycobacterial gene amplification of DNA extracted from water samples (Umkhanyakude District, KwaZulu-Natal, South Africa) with the target gene, amplicon size, and cycling conditions indicated.

Target Gene	Amplicon Size (bp)^a^	Annealing Temperature (°C)^b^	Elongation Time	Reference
*16s*	1,500	65	90 s	Leclerc et al. (47)
*hsp65*	439	62.5	30 s	Telenti et al. (36)
*rpoB*	764	64	30 s	Adékambi et al. (35)
MAC *hsp65*	1,621	55	90 s	Turenne et al. (38)
*gyrA*	107	55	30 s	Landolt et al. (37)
*gyrB1*	144	55	30 s	Landolt et al. (37)
*gyrB2*	107	55	30 s	Landolt et al. (37)

^aExpected amplicon size after PCR and visualization following gel electrophoresis on a 1% agarose gel. bThe temperature for the annealing step and duration of the elongation step differed per PCR. Otherwise cycling conditions consisted of an initial denaturing step at 94°C for 1 min, followed by 35 cycles of denaturation at 94°C for 30 s, the selected annealing temperature for 30 s, elongation at 68°C for the selected duration, and a final elongation step of 5 min at 72°C.^

#### 2.2.3 Sanger amplicon sequencing (SAS)

Sanger amplicon sequencing (SAS) was used to screen samples for mycobacterial DNA (36, 48) using *Mycobacterium* genus-specific *hsp65* amplicons, according to Clarke et al. (49), to provide an affordable prescreening option (48). The *hsp65* PCR was briefly performed using the optimized annealing temperature and elongation times (Table 1). Amplicons were sent for post-PCR clean-up and Sanger sequencing at the Stellenbosch University Central Analytical Facility (CAF, Stellenbosch, South Africa). Sanger sequences were aligned, and consensus sequences were produced using BioEdit Sequence Alignment Editor (version 7.7, Tom Hall, CA, USA). Consensus sequences were compared to the National Centre for Biotechnology Information (NCBI) database with the Basic Local Alignment Search Tool for Nucleotides (BLASTn) program (50).^1^ The percentage coverage (P_C_) and identity match (P_IM_) between Sanger sequences and the NCBI database were recorded. A P_C_ and P_IM_ ≥ 80% with an *hsp65* sequence from a known mycobacterial genome was required for genus-level identification. If no sequences met these criteria, the DNA sample was not used for downstream analysis. If the sequence also matched a known mycobacterial species, including non-tuberculous mycobacteria (NTMs) or MTBC with P_IM_ ≥ 90%, identification was reported at the species level. If sequences from a DNA sample matched MTBC at the species level, the sample was considered positive for MTBC. Since MTBC ecotypes or strains cannot be distinguished based on their *hsp65* amplicon sequences, samples with MTBC DNA were further characterized as described in Section 2.3.2.

#### 2.2.4 Oxford Nanopore Technologies targeted next-generation sequencing (ONT tNGS)

To confirm the presence of mycobacterial species detected by *hsp65* SAS and further characterize the mycobacteriome, additional amplicons (*hsp65*, *rpoB*, MAC *hsp65*, and *gyrase* PCRs) from selected samples were sequenced using ONT tNGS. Amplicons were pooled for each sample in equal molar concentrations (200 fmol), then end-repaired and individually barcoded (39). Briefly, the pooled PCR amplicons (*hsp65*, *rpoB*, MAC *hsp65*, *gyrB1*, and *gyrB2*) from each sample received the same barcode using the Native Barcoding Kit v14 (ONT), Blunt/TA Ligase Master Mix, Next Ultra II End Repair/dA Module, and Quick Ligation Module (all from New England Biolabs). Unique barcode numbers 17–73 were used for samples, while barcode 74 was added to a no-DNA control. Due to the similar lengths of target sequences, *gyrA* amplicons were barcoded separately from the other amplicons (barcoded Nos. 19, 21, and 39) using independent barcodes (Nos. 75, 76, and 77). Barcoded amplicons from all samples were pooled into a single library, native adapters ligated, loaded onto a single R10.4.1 flow cell (>1,250 pores), and sequenced using the MinION mk1C device (ONT). The barcodes’ base-calling, demultiplexing, and trimming were performed using Guppy version 6.4.6, with the high-accuracy option selected (51). Quality control and filtering reads with a Q score of <12 were performed using nanoq version 0.10.0 (52), FastQC version 0.11.9, and pycoQC version 2.5.0.23 (53). Reference-free read sorting was performed using the amplicon sorter tool version 2023-06-19 (54). A total of 200,000 randomly chosen reads with lengths between 50–2000 bp were selected for each barcode. Consensus sequences were grouped based on amplicon size and similarity, and relative abundancies (P_RA_) ≥ 1% retrieved, based on the representative pool of reads analyzed. ABRicate^2^ was applied to screen consensus sequences against customized databases for each target and generate summary report files, according to Ghielmetti et al. (39). The distribution of consensus sequences generated per target gene was visualized using the R package ggplot2 (55). Consensus sequences with P_C_ and P_IM_ < 90% (based on comparison to sequences from the in-house database) were annotated as unclassified. Consensus sequences with P_C_ and P_IM_ ≥ 90%, compared to the sequences from known mycobacterial genomes, were classified to genus level. Consensus sequences with P_C_ ≥ 90% and P_IM_ between 97–99% compared to sequences from a known mycobacterial species were manually inspected using BLASTn (NCBI) before the mycobacterial species with the highest identity match was assigned. However, consensus sequences with P_C_ ≥ 90% and P_IM_ ≥ 99% were assigned as NTMs or MTBC without manual inspection. If the mycobacterial species in a sample was identified as MTBC, the sample was considered positive for MTBC, and DNA was used for downstream characterization according to Section 2.3.2. If no species could be assigned, identification remained at the genus level.

### 2.3 Detection and characterization of MTBC DNA

#### 2.3.1 Detecting MTBC with the GeneXpert® MTB/RIF ultra (GXU) qPCR assay

The GeneXpert® MTB/RIF Ultra (GXU) qPCR assay was selected as an independent method to screen environmental samples for the presence of MTBC DNA, despite being optimized primarily for human sputum samples (56). As previously described, one 10 mL aliquot was used per sample for GXU (57). Each aliquot was centrifuged at 1,000 rcf for 5 min in an Eppendorf 5810R centrifuge to pellet large sediment particles. Notably, 1 mL of supernatant was collected at the interface above the sediment pellet and transferred to a 5 mL tube, after which an equal volume of GXU sample reagent (1 mL) was added. Supernatant samples were vortexed for 10 s before and after a 10 min incubation at room temperature (20–22°C), loaded into a GXU cartridge (Cepheid), placed in the GeneXpert® IV instrument (Cepheid), and analyzed according to manufacturer’s guidelines. The GXU test outputs indicated semiquantitative MTBC DNA levels. In this study, the GXU was repeated if samples returned INVALID/ERROR. The MTB NOT DETECTED output was reported as a negative result (i.e., neither IS*6110* nor IS*1081* amplified). The levels of MTB detected (very low/low/medium/high) were based on preprogrammed *rpoB* cycle threshold (CT) values: very low (Ct > 28), low (Ct 22–28), medium (Ct 16–22) or high (Ct < 16). If MTB was not detected with *rpoB* probes but with IS*6110* and IS*1081*, rifampicin resistance could not be established, and the GXU output was MTB TRACE DETECTED. In this study, MTB TRACE readouts were considered an MTBC positive result since the assay cannot distinguish between MTBC ecotypes or strains. If MTBC DNA was detected, the sample was considered positive for MTBC, and DNA was extracted and used for downstream characterization according to Section 2.3.2. If no MTBC was detected, the sample was not further investigated.

#### 2.3.2 Characterization of MTBC

If GXU, *hsp65* SAS, or ONT tNGS detected MTBC DNA, the site was considered positive, and MTBC DNA was further evaluated to determine which MTBC ecotype (*Mycobacterium africanum*, *M. bovis*, *M. bovis* BCG, *Mycobacterium canettii*, *Mycobacterium caprae*, *Mycobacterium microti*, and *M. tuberculosis*) was present using three PCR-based methods. First, after ONT tNGS (Section 2.2.4), *gyrase* (*gyrA*, *gyrB1*, and *gyrB2*) consensus sequences were compared to the in-house database containing *gyrase* gene regions from known MTBC ecotypes. If the MTBC-specific SNPs, described by Landolt et al. (37), were present and there was a match (P_C_ and P_IM_ ≥ 99%) with the ecotype *M. bovis*, the sample was considered positive for *M. bovis* DNA. Second, the genomic regions of difference (RD) PCR were used to characterize MTBC, according to Warren et al. (58). Briefly, PCR was used to amplify RD1, RD4, RD9, and RD12 gene regions, and amplicon presence or absence was visualized using gel electrophoresis. If the presence or absence of RD amplicons was consistent with that of *M. bovis*, *M. bovis* DNA was considered detected in that sample. Finally, spacer oligonucleotide typing (spoligotyping), performed according to Kamerbeek et al. (59), was used to identify MTBC ecotypes and strains. Briefly, spacer sequences within the direct repeat (DR) gene region were amplified using biotinylated primers and hybridized to synthetic oligonucleotides of known spacer sequences on a membrane. The spacers were visualized on X-ray film and the pattern translated into an octal code. If the spoligotyping pattern/code matched that of a known MTBC ecotype, such as *M. bovis*, *M. bovis* DNA was considered detected. Multiple spoligotyping patterns/codes may be identified as *M. bovis* but differ slightly, they can be used to differentiate *M. bovis* strains if pattern resolution is sufficient. Positive control (*M. bovis*) DNA and a non-template control were included for all three methods. The detection of *M. bovis* with any of the three methods (*gyrase* ONT tNGS, RD-PCR, and spoligotyping) resulted in the sample being considered positive for *M. bovis*. Additionally, analyses of the *gyrB* sequences and RD1 amplicon presence facilitated the differentiation of *M. bovis* from *M. bovis* BCG (58, 60). If the MTBC ecotype could not be identified, the sample was considered to contain uncharacterized MTBC DNA.

### 2.4 Statistical analysis

The detection of MTBC DNA using *hsp65* SAS or ONT tNGS was evaluated compared to the GXU, which is widely used for MTBC detection and is an independent PCR method (61). The percentage observed agreement (p_o_) was calculated for all 63 sites with INVALID/ERROR GXU results considered as negative for MTBC. The chance agreement (p_e_) was calculated based on column and row sums with the equation (proportion of Test A that is positive for MTBC × proportion of Test B that is positive for MTBC) + (proportion of Test A that is negative for MTBC × proportion of Test B that is negative for MTBC). The equation *K* = (*p*_o_ – *p*_e)_/(1 – *p*_e_) was then used to calculate Cohen’s kappa statistic (62, 87). The 95% confidence interval (CI) was calculated in GraphPad Prism version 10 (GraphPad Software, Boston, MA, USA). A scale used by Landis and Koch (63) was used for interpretation agreement as absent (values ≤0), slight (0.01–0.20), fair (0.21–0.40), moderate (0.41–0.60), substantial (0.61–0.80), or near perfect (0.81–1.00). Cohen’s kappa statistics were also used to evaluate the detection of *M. bovis* DNA using ONT tNGS or RD-PCR compared to spoligotyping according to the above methodology.

After MTBC DNA was detected with GXU, SAS, and ONT tNGS, the P_RA_ or CT values of samples that tested positive with all three methods were compared with samples that tested positive based on one or two methods. To this end, a one-way analysis of variance (ANOVA) using Excel version 16 and the XLSTAT add-in (Microsoft Office, Microsoft Corporation, Redmond, WA, USA) was used.

## 3 Results

### 3.1 DNA extraction, PCR, and sanger amplicon sequencing (SAS)

**Figure 1:**
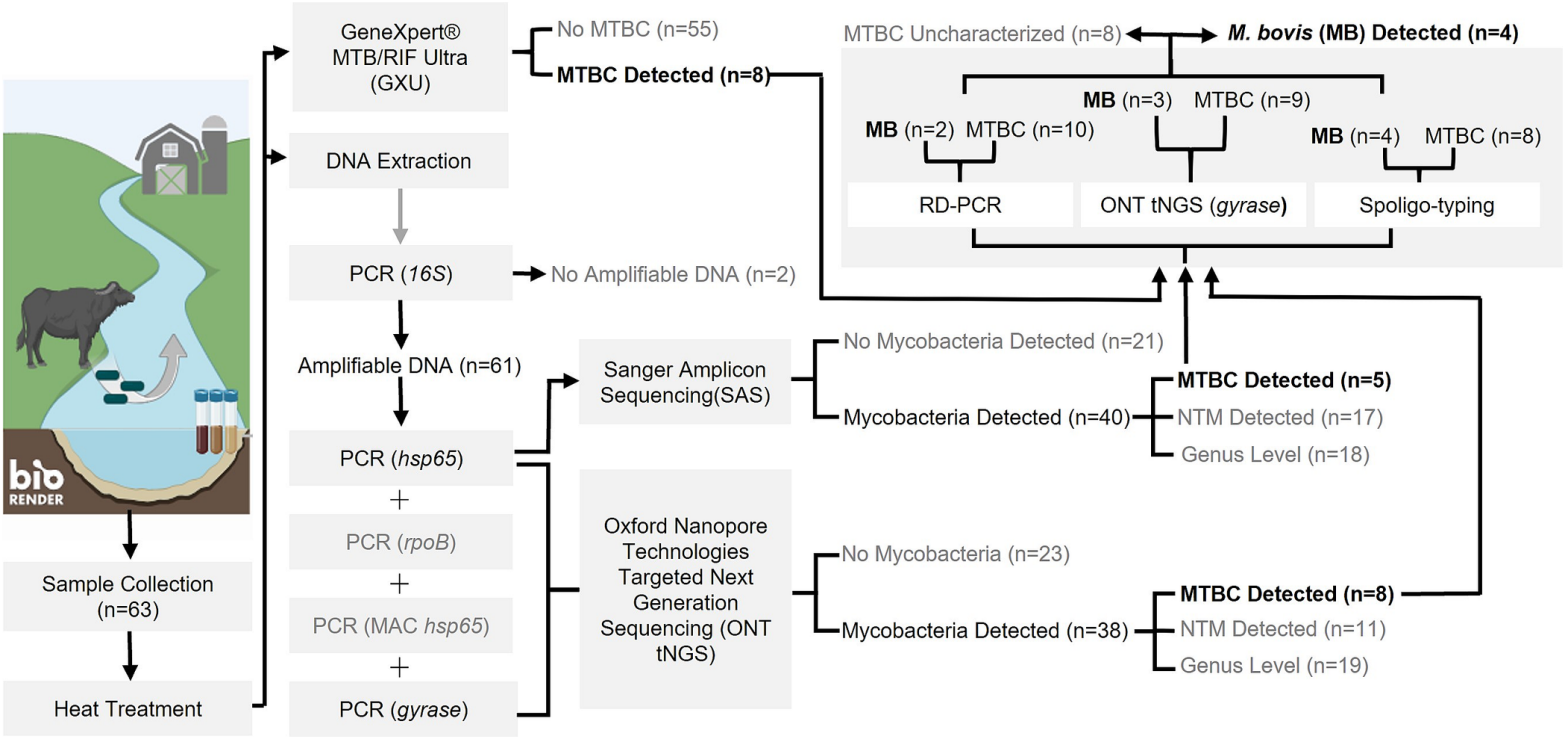
Flowchart outlining study methods and outcomes for PCR-based detection of *Mycobacterium tuberculosis* complex (MTBC) DNA and characterization as *M. bovis* from water sources in the Umkhanyakude District, KwaZulu-Natal, South Africa. Numbers in parentheses represent the number of sample sites tested and results at each step. Steps which did not result in MTBC detection or warrant further downstream analysis are indicated in grey text. Created in BioRender. Matthews, M. (2025) https://BioRender.com/t74p898.

**Figure 2:**
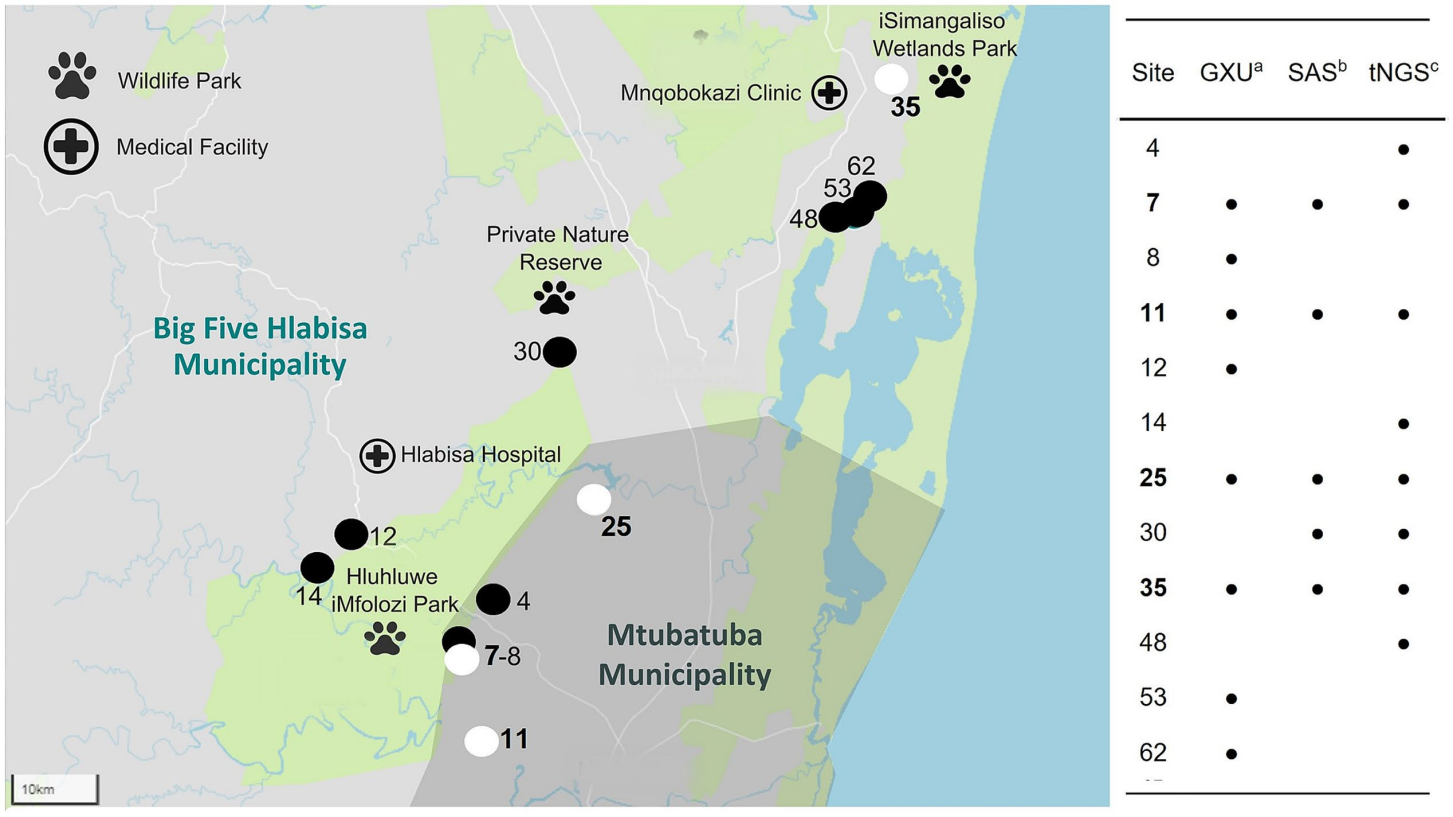
Map of 12 water sample sites (out of 63) where *Mycobacterium tuberculosis* complex (MTBC) DNA was detected, including 4 sites identified to contain *Mycobacterium bovis* (site number highlighted in bold) DNA within the Umkhanyakude District, KwaZulu-Natal, South Africa. Detection was based on results from GeneXpert® MTB/RIF Ultra (GXU)^a^, qPCR assay, Sanger amplicon sequencing (SAS)^b^ of *hsp65* PCR amplicons, and Oxford Nanopore Technologies (ONT) targeted next-generation sequencing (tNGS)^c^ of *hsp65* and *gyrase* PCR amplicons. Sites with MTBC DNA detected by any method are shown with the site number and a black location pin on the map. If MTBC DNA could be characterized as *M. bovis*, the site was marked with a white location pin on the map.

Sample DNA was extracted from 63 sites and screened for mycobacterial species, including MTBC. According to Qubit results, an average of 576 ng/μL (SD: 221 ng/μL) of DNA was extracted. Intact DNA was observed using gel electrophoresis. Although multiple samples were taken from some sites, the results were summarized per site to aid interpretation (Figure 1; Supplementary Table S1). The DNA from all 63 sites, except two (sites 2 and 53), could be amplified with *16S* PCR and, therefore, did not contain PCR inhibitors. The *Mycobacterium* genus-specific PCR (*hsp65*) and SAS detected the presence of mycobacteria in samples from 40 sites. Of these, mycobacterial DNA could be identified at the mycobacterial complex or species level for 23 sites. Samples from 5 of these sites (7, 11, 25, 30, and 35) had detectable MTBC DNA (Figure 2; Table 2), while 10 sites had sequences matching MAC DNA. In addition, a variety of other NTM species were identified, including *Mycobacterium canariasense/cosmeticum*, *Mycobacterium crocinum*, *Mycobacterium madagascariense*, *Mycobacterium novocastrense*, *Mycobacterium parmense*, *Mycobacterium saskatchewanense, Mycobacterium parafortuitum*, *Mycobacterium paraense*, *Mycobacterium vaccae*, and *Mycobacterium nebraskense*.

**Table 2:** Multiple PCR-based methods were used to detect and differentiate *Mycobacterial tuberculosis* complex (MTBC) DNA extracted from water sources in Umkhanyakude District, KwaZulu-Natal, South Africa.

Site Information^a^			MTBC Detection							MTBC Differentiation			*M. bovis* confirmed^h^
			GXU^b^		SAS (*hsp65*)^c^			ONT tNGS (*hsp65*)^d^		ONT tNGS (*gyrase*)^e^	RD-PCR^f^	Spoligo-typing^g^	
No.	LAT	LONG	Result	CT	Result	P_C_	P_IM_	Result	P_RA_	Result	Result	Result	
4	−28,268	32,037	NEG	ND	NEG	0%	0%	**POS**	2%	ND	ND	ND	No
7	−28,291	31,995	**POS**	23.9	**POS**	**100%**	**99%**	**POS**	**67**%	**M. bovis**	**M. bovis**	**M. bovis**	**Yes**
8	−28,302	31,995	**POS**	34	NEG	0%	0%	NEG	0%	ND	ND	ND	No
11	−28,359	31,998	**POS**	28.3	**POS**	**95%**	**91%**	**POS**	**46**%	**M. bovis**	**M. bovis**	**M. bovis**	**Yes**
12	−28,180	31,868	**POS**	30.3	NEG	0%	0%	NEG	0%	ND	ND	ND	No
14	−28,212	31,829	NEG	ND	NEG	0%	0%	**POS**	5%	ND	ND	ND	No
25	−28,144	32,150	**POS**	26.9	**POS**	**98%**	**98%**	**POS**	**78**%	**M. bovis**	ND	**M. bovis**	**Yes**
30	−27,993	32,109	NEG	ND	**POS**	**99%**	**100%**	**POS**	96%	ND	ND	ND	No
35	−27,717	32,494	**POS**	30.7	**POS**	**100%**	**100%**	**POS**	**100**%	ND	ND	**M. bovis**	**Yes**
48	−27,858	32,429	INV	ND	NEG	0%	0%	**POS**	1%	ND	ND	ND	No
53	−27,853	32,454	**POS**	35	NEG	0%	0%	NEG	0%	ND	ND	ND	No
62	−27,804	32,450	**POS**	25.5	NEG	0%	0%	NEG	0%	ND	ND	ND	No

NEG, negative; POS, positive; INV, Invalid; ND, not determined; CT, cycle threshold; P_C_, percentage coverage; P_IM_, percentage identity match; P_RA_, percentage relative abundance. Samples from 12 sites^a^ had detectable MTBC DNA^b,c,d^. Extracted DNA from these samples underwent further tests to speciate MTBC^e,f,g^. *Mycobacterium bovis* (*M. bovis*) was confirmed in samples from four sites, while samples from eight sites contained undifferentiated MTBC DNA. ^a^The site information including site number (No.) and location in latitude (LAT) and longitude (LONG) indicated to three decimal points. ^b^Samples were screened using the GeneXpert® MTB/RIF Ultra (GXU) qPCR assay. If GXU detected MTBC DNA, the sample was considered positive, and the cycle threshold (CT) value was recorded. If no gene targets were detected, the sample was considered negative. If the GXU internal control failed, the result was considered ‘invalid’. ^c^Extracted sample DNA underwent *hsp65* PCR and Sanger amplicon sequencing (SAS). Sequences were compared to the National Centre for Biotechnology Information (NCBI) database. If the sequence had a percentage coverage (P_C_) and identity match (P_IM_) ≥ 90% with known MTBC genomes, the sample was considered MTBC positive. If the sequences were below this threshold, the sample was considered negative for MTBC DNA. ^d^In parallel with SAS, *hsp65* amplicons were sequenced using Oxford Nanopore Technologies targeted next generation sequencing (ONT tNGS) and compared to an in-house curated database. Sequences that matched MTBC had P_C_ and P_IM_ values of 100%. The relative abundance (P_RA_) of MTBC sequences was indicated as a percentage of the total reads assigned per sample site. If no reads matched MTBC above the threshold (P_C_ ≥ 90% and P_IM_ ≥ 97%), the sample was considered negative for MTBC. ^e^The ONT tNGS of *gyrase* (*gyrA, gyrB1* and *gyrB2*) amplicons and comparison to the in-house database was used to differentiate MTBC. If MTBC specific SNPs (37) were present and sequence matched known *M. bovis* genomes*, M. bovis* was considered detected. In this study, *M. bovis* positive sequences had P_C_ and P_IM_ values of 100%. If no MTBC ecotype could be assigned, the MTBC differentiation result was considered not determined ‘ND’. ^f^Region of difference (RD) PCR was used to differentiate MTBC, according to Warren et al. (58). If the presence/absence of RD1, RD4, RD9 and RD12 amplicons was consistent with *M. bovis*, *M. bovis* was considered detected. If no MTBC ecotype could be assigned, the MTBC differentiation result was considered not determined ‘ND’. ^g^Spacer oligonucleotide typing (spoligo-typing) was used to differentiate MTBC, according to Kamerbeek et al. (59). If the MTBC spoligo-type was consistent with known *M. bovis* patterns, *M. bovis* was considered detected. If no MTBC ecotype could be assigned, the spoligo-type result was not determined ‘ND’. ^h^Detection of *M. bovis* DNA, based on a positive result in *gyrase* ONT tNGS, RD-PCR or spoligo-typing, was used to confirm the presence of *M. bovis* at the site. Positive results for MTBC/*M. bovis* are indicated in bold.

### 3.2 Oxford nanopore targeted next-generation sequencing (ONT tNGS)

Since environmental samples were expected to have a complex mycobacteriome, multiple mycobacterial PCR amplicons (*hsp65*, *rpoB*, MAC *hsp65*, and *gyrase*) underwent ONT tNGS to evaluate the P_RA_ and diversity of the mycobacterial species present. Barcoded samples had an average of 202,544 reads (SD: 85,003) and an average read quality score of 16 (SD: 0.39). The ONT tNGS of the *hsp65* gene target indicated the presence of mycobacterial DNA at 38 sites, including NTMs (MAC, *M. crocinum*, *M. canariasense/cosmeticum*, *M. madagascariense*, *M. novocastrense*, *M. parmense*, and *M. saskatchewanense*) at 11 sites, and MTBC in samples from 8 sites (Nos. 4, 7, 11, 14, 25, 30, 35, and 48). The P_RA_ of MTBC DNA ranged from 1 to 100% (Table 2; Supplementary Table S1). All five MTBC-positive sites identified by screening with *hsp65* PCR and SAS (Nos. 7, 11, 25, 30, and 35) were confirmed by ONT tNGS (Figure 2; Table 2; Supplementary Table S1). In contrast, ONT tNGS of the *rpoB* gene target detected mycobacterial DNA at genus level at 30 sites, NTMs to species level at four sites (Nos. 22, 34, 48, and 52), but no MTBC DNA. After ONT tNGS, MAC *hsp65* consensus sequences could not be classified to the species level using a ≥ 90% P_IM_ threshold and were not differentiated further. Based on these results for *rpoB* and MAC *hsp65* ONT tNGS, they were excluded from Supplementary Table S1.

### 3.3 Detecting MTBC DNA with the GXU, SAS, or ONT tNGS

As an independent rapid screening method for MTBC DNA detection in this pilot study, the GXU was performed on a separate sample aliquot from the 63 sites (Figure 1; Supplementary Table S1). Samples from four sites (Nos. 34, 39, 48, and 51) had INVALID/ERROR results, even after GXU was repeated. Of the remaining samples tested, the majority had no detectable MTB. However, eight sample sites (Nos. 7, 8, 11, 12, 25, 35, 53, and 62) had an MTB TRACE detected result, which was considered positive for MTBC in this study. The CT value for positive samples ranged between 23.9 to 35 (Table 2; Supplementary Table S1). A comparison of MTBC DNA detection results between GXU, *hsp65* SAS, and ONT tNGS is shown in Figure 2 and Table 2. There was moderate agreement between GXU and SAS (kappa statistic of 0.57) or ONT tNGS results (kappa statistic of 0.43), respectively (Supplementary Table S2). The DNA from four sites (Nos. 7, 11, 25, and 35) tested positive using all three methods and showed higher median P_RA_ (72.5%) and lower CT values (27.6) compared to samples that were positive in only one or two tests. The differences were, however, not significant (*p* > 0.05).

### 3.4 Characterization of MTBC

To further speciate MTBC DNA detected at 12 sites (Nos. 4, 7, 8, 11, 12, 14, 25, 30, 35, 48, 53, and 62) based on GXU, *hsp65* SAS or ONT tNGS, three PCR-based methods were used in parallel (Figures 1, 2; Table 2). At four sites (Nos. 7, 11, 25, and 35), *M. bovis* DNA was confirmed to be present based on spoligotyping. Although the spoligotyping pattern was consistent with *M. bovis*, the blot was too faint to assign a strain or spoligotype specific code conclusively. Using RD-PCR and *gyrase* ONT tNGS, *M. bovis* was detected at two (Nos. 7 and 11) and three (Nos. 7, 11, and 25) sites, respectively. Moreover, there was moderate (kappa statistic 0.57) and substantial (kappa statistic 0.80) agreement between spoligotyping and RD-PCR and ONT tNGS results, respectively (Supplementary Table S3). Overall, *M. bovis* DNA was confirmed in 4 sites (Nos. 7, 11, 25, and 35) with one or more PCR based methods, while MTBC DNA was considered detected but could not be characterized further at the remaining eight sites (Nos. 4, 8, 12, 14, 30, 48, 53, and 62).

## 4 Discussion

In this study, GXU, *hsp65* SAS, and ONT tNGS were combined to enhance culture-independent detection of MTBC DNA from water sources in KZN. Further differentiation of MTBC was based on RD-PCR, spoligotyping, and *gyrase* ONT tNGS. Environmental samples from 12 sites at livestock-wildlife-human interfaces were found to contain MTBC DNA, with four sites confirmed to contain *M. bovis* DNA, in selected areas with high burdens of human and animal TB (43). Although eight samples with MTBC could not be identified at the ecotype level, it is possible these sites were contaminated by either *M. bovis* or *M. tuberculosis* since both animals and human rural communities used the locations. The presence of either MTBC ecotype is essential for understanding the potential role of the environment in TB epidemiology (13).

The detection of *M. bovis*/MTBC DNA in environmental samples is an arduous process but has been successful in the United Kingdom, France, and Portugal (11, 33, 64). Although a previous attempt to find environmental *M. bovis* in South Africa was unsuccessful (65), advances in molecular tools improved this study’s detection feasibility. The presence of *M. bovis* DNA in shared water sources in KZN suggests that the source of contamination could be infected local livestock or wildlife and, less likely, human populations. This is not surprising since epidemiological links have been shown between environmental *M. bovis* and infected local animal populations in a previous study (33). Moreover, the porous boundaries of HiP facilitate interactions at livestock-wildlife-human interfaces, leading to potential spillover between the park and local communities (43, 46). In addition, sharing untreated water sources by people and animals could result in MTBC contamination and increased infection risk. Increased MTBC surveillance in humans, animals, and the environment would provide a more comprehensive approach and is especially important due to the presence of other human diseases such as human immunodeficiency virus (HIV)/acquired immunodeficiency syndrome (AIDS) (43, 66). However, managing MTBC environmental contamination would entail enforcing disease management and sanitation practices that limit risk. For example, maintaining barriers between wildlife and domestic animals, bactericidal water treatments, and timely disposal of animal excrement and carcasses (8, 67, 68, 88). Therefore, further investigations are needed to understand the complex TB epidemiology in these systems and inform health interventions (13, 42) to minimize risk.

In this study, MTBC DNA was detected more frequently with GXU or ONT tNGS than with SAS. However, the GXU and ONT tNGS results were discordant at 50% of sites. According to Verma et al. (69), GXU detected environmental MTBC at “trace” levels, which is beyond the limit of detection of the *rpoB* probes but not IS*6110*/*1081* probes. This complicates interpretation and means positive GXU results confirmed by ONT tNGS are likely more reliable (70). Similarly, discordant GXU negative results could be due to platform-specific limitations when using complex samples, including PCR inhibition. For example, sample site 30 was GXU negative, although strong sequencing signals were observed with SAS and ONT tNGS. This highlights the potential for false negative GXU results and the importance of using a multi-modal approach. As with GXU, the detection of MTBC DNA varied across (*hsp65*, *rpoB*, and *gyrase*) gene targets when evaluating ONT tNGS results. As shown in previous research, variation in the *hsp65* gene region appeared more informative than *rpoB* for species or complex-level detection of mycobacteria (71). Furthermore, MTBC detection based on the *hsp65* sequences only correlated with detection and characterization based on *gyrase* sequences in 25% of sites. The differences in gene target performance could be explained by sample composition, amplicon size, and nucleotide content, which affect PCR and tNGS efficiency (72). Future assay validation should, therefore, determine sensitivity per gene target using a dilution series of target DNA within different sample matrices. Due to the variations in performance between gene targets and sequencing methods, multiple molecular assays should be used in parallel rather than as a simple screening hierarchy for reliable environmental MTBC surveillance.

As NTMs are ubiquitous in the environment (73), it was unsurprising that *hsp65* SAS and ONT tNGS identified several NTMs. Unlike SAS, ONT tNGS could provide P_RA_ estimates and detect NTM with or without MTBC. Of the NTMs detected, MAC, *M. madagascariense*, and *M. nebraskense* have previously been reported in water and soil samples from KZN (74), as well as from livestock, wildlife, and people in this province (41, 44, 74). Unfortunately, differentiation based on MAC *hsp65* was impossible due to low sequence quality and poor homology with known MAC sequences in the database. Other gene targets, such as IS*900* and IS*901*, should be investigated for differentiating MAC strains in the future studies (19, 75). Of the NTMs detected, some were not previously reported in KZN. These findings provide useful information for comparison with future human clinical or veterinary mycobacterial isolates from these communities. Although this study identified NTMs and MTBC, incorporating a mycobacterial mock community (76) in the future studies would facilitate a more standardized comparison between mycobacteriome projects across different environments.

Although *M. bovis* detection could be confirmed by RD-PCR, spoligotyping, or *gyrase* ONT tNGS, these methods could not differentiate the MTBC ecotype in 8 of the 12 positive samples. Therefore, it was impossible to determine if contamination was due to MTB or *M. bovis*. In addition, the *M. bovis* spoligotyping could not be assigned, which could have provided an epidemiological link with infected animal populations in the area (39, 43). In samples where MTBC was detected, but the ecotype could not be assigned, it was likely that DNA from *M. bovis* or other MTBC was present but at levels too low for differentiation (77). Therefore, different approaches, such as magnetic bead capture techniques, should be explored to enrich MTBC in samples pre- or post-DNA extraction (33, 78). Moreover, if the methods by Pereira et al. (33) were used, the diversity of *M. bovis* strains based on SNP diversity (33) rather than spoligotyping could be explored. The complex nature and heterogeneity of mycobacterial species present in environmental samples could also have led to challenges in attributing a single MTBC ecotype using the techniques employed in this study, due to the 99.9% genetic similarity of members in this group (79).

A limitation of this study was that although the P_RA_ of MTBC ONT tNGS *hsp65* sequences and GXU CT values provided a crude estimate of MTBC concentrations per sample, the effect of environmental conditions and animal density could not be explored due to the limited sampling time (4 days), resources and data availability. According to a study by Courtenay et al. (64), the quantity of *M. bovis* DNA alone in soil may provide some insights into the infection status of animal populations, as shown in a study of local badgers and proximal cattle farms. In contrast, a study by Martínez-Guijosa et al. (80) found detection of environmental *M. bovis* DNA was not correlated with host animal prevalence or disease. Still, it was associated with certain environmental conditions. These variables should be explored in the future studies, especially as research has predominantly focused on environmental NTMs, or MTBC in general (13, 81, 82).

A significant limitation of this study was the requirement (from the South African Department of Agriculture) to heat-inactivate samples before transport, which precluded mycobacterial culture. This would have provided additional confirmation of viable mycobacteria present and potentially increased detection of MTBC since the expected paucibacillary nature of these samples may have been below the limits of detection (15). However, despite heat treatment, high quantities of amplifiable DNA could be extracted, amplified, and sequenced. Moreover, in this study, ONT tNGS was moderately and substantially comparable to more traditional molecular assays for MTBC and *M. bovis* detection. The amplicon-targeted approach was chosen as it was more sensitive for culture-independent detection of specific environmental microorganisms compared to shotgun sequencing (83, 84). The disadvantage is that using PCR for target enrichment prior to tNGS, may introduce bias, producing results not reflective of the original mycobacteriome (85). Additional culture-independent techniques that should be explored in the future studies to quantify viable *M. bovis* preferentially could include fluorescent labeling and sorting of MTBC cells with flow cytometry (86).

Despite the limitations of this study, *M. bovis* and MTBC DNA were successfully detected in environmental samples collected from a high TB burden area within KZN, South Africa, suggesting possible contamination by infected animal and or human populations. Environmental sources of MTBC should be investigated further to improve our understanding of TB epidemiology in complex multihost systems.

## Data Availability

Raw ONT tNGS reads from sites where mycobacterial DNA was detected as per Supplementary Table S1 were deposited in GenBank (Bioproject ID: PRJNA1185125).

## Footnotes

^1^ https://blast.ncbi.nlm.nih.gov

^2^ https://github.com/tseemann/abricate
