# Supplementary material for: Evidence of Mycobacterium bovis DNA in shared water sources at livestock–wildlife–human interfaces in KwaZulu-Natal, South Africa
Source: Front Vet Sci. 2025 Feb 28;12:1483162. doi: 10.3389/fvets.2025.1483162 (PMC11907651; doi:10.3389/fvets.2025.1483162)
Supplement: Supplementary file 1 [file Data_Sheet_1.DOCX]

Supplementary Material

Table S1. Summary of results for 63 water sampling sites^a^ screened for *Mycobacterium tuberculosis* complex (MTBC) DNA, based on DNA extraction and *hsp65* PCR^b^ followed by Sanger amplicon sequencing (SAS)^c^ or Oxford Nanopore (ONT) targeted next generation sequencing (tNGS)^d^. Results from *hsp65* PCR ONT tNGS were used to calculate relative abundance of mycobacterial species/complex DNA. The GeneXpert^®^ MTB/RIF Ultra (GXU)^e^ qPCR assay was used as an independent method of MTBC DNA detection.

| Site Information^a^ | | | | *16S* PCR^b^ | *hsp65* SAS^c^ | *hsp65* ONT tNGS^d^ | | GXU^e^ | |
| --- | --- | --- | --- | --- | --- | --- | --- | --- | --- |
| Number | Date | LAT | LONG |  |  | Result | MTBC P_RA_ (%) | Result | CT Value |
| 1 | 24/04/2023 | -28,268 | 32,037 | Amplified | No Mycobacteria detected | No Mycobacteria detected | 0% | No MTBC | No CT |
| 2 | 24/04/2023 | -28,253 | 32,059 | No amplicon | Not included | Not included | Not included | No MTBC | No CT |
| 3 | 24/04/2023 | -28,243 | 32,034 | Amplified | No Mycobacteria detected | No Mycobacteria detected | 0% | No MTBC | No CT |
| 4 | 24/04/2023 | -28,244 | 32,033 | Amplified | *M. kansasii* | **MTBC** and *Mycobacterium* sp. | 2% | No MTBC | No CT |
| 5 | 24/04/2023 | -28,254 | 32,024 | Amplified | No Mycobacteria detected | No Mycobacteria detected | 0% | No MTBC | No CT |
| 6 | 24/04/2023 | -28,293 | 31,997 | Amplified | No Mycobacteria detected | No Mycobacteria detected | 0% | No MTBC | No CT |
| 7 | 24/04/2023 | -28,291 | 31,995 | Amplified | **MTBC** | **MTBC** and *Mycobacterium* sp. | 67% | **MTBC** | 23.9 |
| Site Information^a^ | | | | *16S* PCR^b^ | *hsp65* SAS^c^ | *hsp65* ONT tNGS^d^ | | GXU^e^ | |
| Number | Date | LAT | LONG |  |  | Result | MTBC P_RA_ (%) | Result | CT Value |
| 8 | 24/04/2023 | -28,302 | 31,995 | Amplified | *Mycobacterium* sp. | *Mycobacterium* sp. | 0% | **MTBC** | 34.0 |
| 9 | 24/04/2023 | -28,352 | 31,996 | Amplified | No Mycobacteria detected | No Mycobacteria detected | 0% | No MTBC | No CT |
| 10 | 24/04/2023 | -28,359 | 32,005 | Amplified | No Mycobacteria detected | No Mycobacteria detected | 0% | No MTBC | No CT |
| 11 | 24/04/2023 | -28,359 | 31,998 | Amplified | **MTBC** | **MTBC***, M. madagascariense* and *Mycobacterium* sp. | 46% | **MTBC** | 28.3 |
| 12 | 25/04/2023 | -28,180 | 31,868 | Amplified | *Mycobacterium* sp. | *Mycobacterium* sp. | 0% | **MTBC** | 30.3 |
| 13 | 25/04/2023 | -28,203 | 31,847 | Amplified | *Mycobacterium* sp. | *Mycobacterium* sp. | 0% | No MTBC | No CT |
| 14 | 25/04/2023 | -28,212 | 31,829 | Amplified | *Mycobacterium* sp. | **MTBC** and *Mycobacterium* sp. | 5% | No MTBC | No CT |
| 15 | 25/04/2023 | -28,198 | 31,800 | Amplified | *Mycobacterium* sp. | *Mycobacterium* sp. | 0% | No MTBC | No CT |
| 16 | 25/04/2023 | -28,200 | 31,790 | Amplified | *Mycobacterium* sp. | *Mycobacterium* sp. | 0% | No MTBC | No CT |
| 17 | 25/04/2023 | -28,177 | 31,727 | Amplified | No Mycobacteria detected | No Mycobacteria detected | 0% | No MTBC | No CT |
| 18 | 25/04/2023 | -28,186 | 31,717 | Amplified | No Mycobacteria detected | No Mycobacteria detected | 0% | No MTBC | No CT |
| Site Information^a^ | | | | *16S* PCR^b^ | *hsp65* SAS^c^ | *hsp65* ONT tNGS^d^ | | GXU^e^ | |
| Number | Date | LAT | LONG |  |  | Result | MTBC P_RA_ (%) | Result | CT Value |
| 19 | 25/04/2023 | -28,193 | 31,781 | Amplified | No Mycobacteria detected | No Mycobacteria detected | 0% | No MTBC | No CT |
| 20 | 25/04/2023 | -28,195 | 31,859 | Amplified | *Mycobacterium* sp. | *Mycobacterium* sp. | 0% | No MTBC | No CT |
| 21 | 25/04/2023 | -28,176 | 31,882 | Amplified | MAC | *Mycobacterium* sp. | 0% | No MTBC | No CT |
| 22 | 25/04/2023 | -28,170 | 31,889 | Amplified | MAC | *M. crocinum* and *Mycobacterium* sp. | 0% | No MTBC | No CT |
| 23 | 26/04/2023 | -28,118 | 32,184 | Amplified | MAC | *M. crocinum* and *Mycobacterium* sp. | 0% | No MTBC | No CT |
| 24 | 26/04/2023 | -28,144 | 32,151 | Amplified | No Mycobacteria detected | No Mycobacteria detected | 0% | No MTBC | No CT |
| 25 | 26/04/2023 | -28,144 | 32,150 | Amplified | **MTBC** | **MTBC** and *Mycobacterium* sp. | 78% | **MTBC** | 26.9 |
| 26 | 26/04/2023 | -28,151 | 32,142 | Amplified | No Mycobacteria detected | No Mycobacteria detected | 0% | No MTBC | No CT |
| 27 | 26/04/2023 | -28,055 | 32,157 | Amplified | No Mycobacteria detected | No Mycobacteria detected | 0% | No MTBC | No CT |
| 28 | 26/04/2023 | -28,030 | 32,156 | Amplified | No Mycobacteria detected | No Mycobacteria detected | 0% | No MTBC | No CT |
| Site Information^a^ | | | | *16S* PCR^b^ | *hsp65* SAS^c^ | *hsp65* ONT tNGS^d^ | | GXU^e^ | |
| Number | Date | LAT | LONG |  |  | Result | MTBC P_RA_ (%) | Result | CT Value |
| 29 | 26/04/2023 | -27,998 | 32,112 | Amplified | No Mycobacteria detected | No Mycobacteria detected | 0% | No MTBC | No CT |
| 30 | 26/04/2023 | -27,993 | 32,109 | Amplified | **MTBC** | **MTBC** and *Mycobacterium* sp. | 96% | No MTBC | No CT |
| 31 | 26/04/2023 | -27,717 | 32,486 | Amplified | No Mycobacteria detected | No Mycobacteria detected | 0% | No MTBC | No CT |
| 32 | 26/04/2023 | -27,717 | 32,487 | Amplified | No Mycobacteria detected | No Mycobacteria detected | 0% | No MTBC | No CT |
| 33 | 26/04/2023 | -27,712 | 32,496 | Amplified | *M. seoulens* | *Mycobacterium* sp. | 0% | No MTBC | No CT |
| 34 | 26/04/2023 | -27,711 | 32,500 | Amplified | *M. parmense* | No Mycobacteria detected | 0% | INVALID | No CT |
| 35 | 26/04/2023 | -27,717 | 32,494 | Amplified | **MTBC** | **MTBC** | 100% | **MTBC** | 30.7 |
| 36 | 26/04/2023 | -27,717 | 32,475 | Amplified | No Mycobacteria detected | No Mycobacteria detected | 0% | No MTBC | No CT |
| 37 | 26/04/2023 | -27,716 | 32,474 | Amplified | *Mycobacterium* sp. | *Mycobacterium* sp. | 0% | No MTBC | No CT |
| 38 | 26/04/2023 | -27,715 | 32,474 | Amplified | *M. seoulens* | *M. parmense and Mycobacterium* sp. | 0% | No MTBC | No CT |
| Site Information^a^ | | | | *16S* PCR^b^ | *hsp65* SAS^c^ | *hsp65* ONT tNGS^d^ | | GXU^e^ | |
| Number | Date | LAT | LONG |  |  | Result | MTBC P_RA_ (%) | Result | CT Value |
| 39 | 26/04/2023 | -27,714 | 32,467 | Amplified | *Mycobacterium* sp. | *M. saskatchewanense* and *Mycobacterium* sp. | 0% | No MTBC | No CT |
| 40 | 26/04/2023 | -27,710 | 32,464 | Amplified | *M. kansasii* | *Mycobacterium* sp. | 0% | No MTBC | No CT |
| 41 | 26/04/2023 | -27,751 | 32,454 | Amplified | MAC and *M. colombiense* | *Mycobacterium* sp. | 0% | No MTBC | No CT |
| 42 | 26/04/2023 | -27,758 | 32,453 | Amplified | *M. kansasii* | *Mycobacterium* sp. | 0% | No MTBC | No CT |
| 43 | 26/04/2023 | -27,786 | 32,431 | Amplified | MAC and *M. anyangense* | *M. saskatchewanense* and *Mycobacterium* sp. | 0% | No MTBC | No CT |
| 44 | 26/04/2023 | -27,784 | 32,433 | Amplified | MAC | *Mycobacterium* sp. | 0% | No MTBC | No CT |
| 45 | 26/04/2023 | -27,788 | 32,437 | Amplified | *Mycobacterium* sp. | *Mycobacterium* sp. | 0% | No MTBC | No CT |
| 46 | 26/04/2023 | -27,796 | 32,451 | Amplified | *Mycobacterium* sp. | *Mycobacterium* sp. | 0% | No MTBC | No CT |
| 47 | 26/04/2023 | -27,801 | 32,442 | Amplified | No Mycobacteria detected | No Mycobacteria detected | 0% | No MTBC | No CT |
| 48 | 27/04/2023 | -27,858 | 32,429 | Amplified | *M. anyangense* | **MTBC** and *Mycobacterium* sp. | 1% | INVALID | No CT |
| 49 | 27/04/2023 | -27,860 | 32,431 | Amplified | No Mycobacteria detected | No Mycobacteria detected | 0% | No MTBC | No CT |
| Site Information^a^ | | | | *16S* PCR^b^ | *hsp65* SAS^c^ | *hsp65* ONT tNGS^d^ | | GXU^e^ | |
| Number | Date | LAT | LONG |  |  | Result | MTBC P_RA_ (%) | Result | CT Value |
| 50 | 27/04/2023 | -27,860 | 32,431 | Amplified | No Mycobacteria detected | No Mycobacteria detected | 0% | No MTBC | No CT |
| 51 | 27/04/2023 | -27,861 | 32,430 | Amplified | *Mycobacterium* sp. | No Mycobacteria detected | 0% | No MTBC | No CT |
| 52 | 27/04/2023 | -27,866 | 32,446 | Amplified | MAC | *M. saskatchewanense* and *Mycobacterium* sp. | 0% | No MTBC | No CT |
| 53 | 27/04/2023 | -27,853 | 32,454 | No amplicon | Not included | Not included | Not included | **MTBC** | 35.0 |
| 54 | 27/04/2023 | -27,851 | 32,449 | Amplified | MAC | *Mycobacterium* sp. | 0% | No MTBC | No CT |
| 55 | 27/04/2023 | -27,888 | 32,456 | Amplified | *M. kubicae* | MAC and *Mycobacterium* sp. | 0% | No MTBC | No CT |
| 56 | 27/04/2023 | -27,892 | 32,455 | Amplified | *Mycobacterium* sp. | *Mycobacterium* sp*.* | 0% | No MTBC | No CT |
| 57 | 27/04/2023 | -27,899 | 32,455 | Amplified | *Mycobacterium* sp. | *M. novocastrense* and *Mycobacterium* sp. | 0% | No MTBC | No CT |
| 58 | 27/04/2023 | -27,866 | 32,460 | Amplified | *Mycobacterium* sp. | *Mycobacterium* sp. | 0% | No MTBC | No CT |
| 59 | 27/04/2023 | -27,859 | 32,462 | Amplified | No Mycobacteria detected | No Mycobacteria detected | 0% | No MTBC | No CT |
| Site Information^a^ | | | | *16S* PCR^b^ | *hsp65* SAS^c^ | *hsp65* ONT tNGS^d^ | | GXU^e^ | |
| Number | Date | LAT | LONG |  |  | Result | MTBC P_RA_ (%) | Result | CT Value |
| 60 | 27/04/2023 | -27,835 | 32,472 | Amplified | MAC and *M. parascrofulaceum* | MAC, *M. saskatchewanense* and *Mycobacterium* sp. | 0% | No MTBC | No CT |
| 61 | 27/04/2023 | -27,850 | 32,437 | Amplified | *Mycobacterium* sp. | *Mycobacterium* sp*.* | 0% | No MTBC | No CT |
| 62 | 27/04/2023 | -27,804 | 32,450 | Amplified | MAC and *M. kubicae* | *M. canariasense/cosmeticum* and *Mycobacterium* sp*.* | 0% | **MTBC** | 25.5 |
| 63 | 27/04/2023 | -27,803 | 32,450 | Amplified | *Mycobacterium* sp. | *Mycobacterium* sp*.* | 0% | No MTBC | No CT |

^a^The site information includes the site number (1-63), date of collection, and location in latitude (LAT) and longitude (LONG). Although multiple samples were taken from some sites, results were summarized per site to facilitate interpretation.

^b^Extracted sample DNA was tested using *16S* PCR to determine if PCR inhibitors were present. If *16S* could be amplified and appropriately sized amplicons were visible after gel electrophoresis, the DNA was used in further PCRs and sequencing. If not, the DNA was not included in downstream assays.

^c^Extracted DNA was amplified with *hsp65* PCR primers, and Sanger amplicon sequenced (SAS). Based on percentage coverage (P_C_) and identity match (P_IM_) with sequences on the National Centre for Biotechnology Information’s (NCBI) database, sequences from each site were identified to genus (P_C_ and P_IM_ ≥ 80%) and/or species level (P_C_ ≥ 80% and P_IM_ ≥ 90%).

^d^Sample DNA identified to contain mycobacterial DNA (based on SAS results), were used in subsequent *hsp65* PCR and Oxford Nanopore Technologies (ONT) amplicon targeted next generation sequencing (tNGS). Sequences were identified to genus (P_C_ and P_IM_ ≥ 90%) or species level (P_C_  ≥ 90% and P_IM_ > 97%) after comparison to an in-house database. The relative abundance (P_RA_) of reads which matched MTBC as a percentage of the total reads assigned per sample is indicated in the second sub column if ≥ 1%.

^e^The GeneXpert^®^ MTB/RIF Ultra (GXU) qPCR assay was to detect *M. tuberculosis* complex (MTBC) DNA in environmental samples. The results are recorded as INVALID, no MTB, or MTB DETECTED in trace/low/medium/high amounts. Since GXU cannot distinguish *M. tuberculosis* from other MTBC, MTB DETECTED is annotated as an MTBC positive result in the table. Cycle threshold (CT) values are included if MTBC DNA was detected in the second sub column.

Table S2. Comparison of detection results for *Mycobacterium tuberculosis* complex (MTBC)^a^ DNA, based on *hsp65* PCR Sanger amplicon sequencing (SAS) or Oxford Nanopore Technologies targeted next generation sequencing (ONT tNGS), with GeneXpert^®^ MTB/RIF Ultra (GXU) qPCR results, using Cohen’s kappa statistic^b^.

|  | GXU Results | |  |  |  | |  |
| --- | --- | --- | --- | --- | --- | --- | --- |
| SAS Results | MTBC^a^ | No MTBC | Row Sum | Cohen’s Kappa^b^ | | Interpretation^c^ | |
| MTBC | 4 | 1 | 5 | 0.57 (CI 0.24 to 0.91) | | Moderate Agreement | |
| No MTBC | 4 | 54 | 58 |  | |  | |
| Column Sum | 8 | 55 | 63 |  | |  | |
|  | GXU Results | |  |  | |  | |
| ONT tNGS Results | MTBC | No MTBC | Row Sum | Cohen’s Kappa^b^ | | Interpretation^c^ | |
| MTBC | 4 | 4 | 8 | 0.43 (CI 0.097 to 0.76) | | Moderate Agreement | |
| No MTBC | 4 | 51 | 55 |  | |  | |
| Column Sum | 8 | 55 | 63 |  | |  | |

^a^The number of sites considered positive for *Mycobacterium tuberculosis* complex (MTBC) DNA was based on sequencing *hsp65* amplicons with Sanger amplicon sequencing (SAS) or Oxford Nanopore Technologies targeted next generation sequencing (ONT tNGS) and comparison to the National Center for Biotechnology Information (NCBI) or an in-house database, respectively. The detection of MTBC DNA with SAS or ONT tNGS was compared to an independent assay, GeneXpert^®^ MTB/RIF Ultra (GXU) qPCR assay.

^b^The Cohen’s kappa statistic was used to compare methods for MTBC detection and was calculated using the equation K= (p_o_ – p_e)_ / (1 – p_e_). The observed proportion of agreement (p_o_) and expected proportion of agreement (p_e_) were calculated as previously reported (McHugh, 2012; Falotico and Quatto, 2015). The 95% confidence interval (CI) was calculated using GraphPad Prism and indicated up to three decimal points.

^c^Agreement was considered absent (values ≤ 0), slight (0.01–0.20), fair (0.21–0.40), moderate (0.41– 0.60), substantial (0.61–0.80) or near perfect (0.81–1.00), based on Cohen’s kappa statistic, according to Landis and Koch (1977).

Table S3. Comparison of detection results for *Mycobacterium bovis* (*M. bovis*)^a^ DNA, based on region of difference (RD) PCR or *gyrase* PCR Oxford Nanopore Technologies targeted next generation sequencing (ONT tNGS), with spacer oligonucleotide typing (spoligo-typing) results, using Cohen’s kappa statistic^b^.

|  | Spoligo-typing Results | |  |  |  | |  |
| --- | --- | --- | --- | --- | --- | --- | --- |
| RD-PCR Results | *M. bovis* ^a^ | No *M. bovis* | Row Sum | Cohen’s Kappa^b^ | | Interpretation^c^ | |
| *M. bovis* | 2 | 0 | 2 | 0.57 (CI 0.08 to 1.00) | | Moderate Agreement | |
| No *M. bovis* | 2 | 8 | 10 |  | |  | |
| Column Sum | 4 | 8 | 12 |  | |  | |
|  | Spoligo-typing Results | |  |  | |  | |
| ONT tNGS Results | *M. bovis^a^* | No *M. bovis* | Row Sum | Cohen’s Kappa^b^ | | Interpretation^c^ | |
| *M. bovis* | 3 | 0 | 3 | 0.80 (CI 0.43-1.00) | | Substantial Agreement | |
| No *M. bovis* | 1 | 8 | 9 |  | |  | |
| Column Sum | 4 | 8 | 12 |  | |  | |

^a^The number of sites that were positive for *Mycobacterium bovis* was based on spacer oligonucleotide typing (spoligo-typing), according to Kamerbeek *et al*. (1997), region of difference (RD) PCR, according to Warren *et al*. (2006), or Oxford Nanopore Technologies targeted next generation sequencing (ONT tNGS) of *gyrase* (*gyrA, gyrB1 and gyrB2*) PCR amplicons, according to Landolt *et al*. (2019). The *gyrase* result was considered positive if amplicon sequences were consistent with *M. bovis,* using an in-house database (coverage and percentage identity ≥ 97%). Likewise, if the spoligo-typing pattern and RD-PCR results were consistent with known *M. bovis* isolates, the sample was considered positive for *M. bovis* DNA. If *M. bovis* or another *Mycobacterium tuberculosis* complex (MTBC) ecotype could not be identified, a sample was considered uncharacterized.

^b^ The Cohen’s kappa statistic was calculated using the equation K= (p_o_ – p_e)_ / (1 – p_e_). The observed proportion of agreement (p_o_) and expected proportion of agreement (p_e_) were calculated as previously reported (McHugh, 2012; Falotico and Quatto, 2015). The 95% confidence interval (CI) was calculated using GraphPad Prism and indicated up to two decimal points.

^c^Agreement was considered absent (values ≤ 0), slight (0.01–0.20), fair (0.21–0.40), moderate (0.41– 0.60), substantial (0.61–0.80) or near perfect (0.81–1.00), based on Cohen’s Kapa statistic, according to Landis and Koch (1977).

**Figure S1**. Photographs of the 63 sampling sites in KwaZulu-Natal, South Africa, from which water margin samples were collected. Photographs per site were organized in order from right to left e.g. sites 1-7 are shown from column A to I on row 1, and sites 8-14 on row 2 column A-I.

**
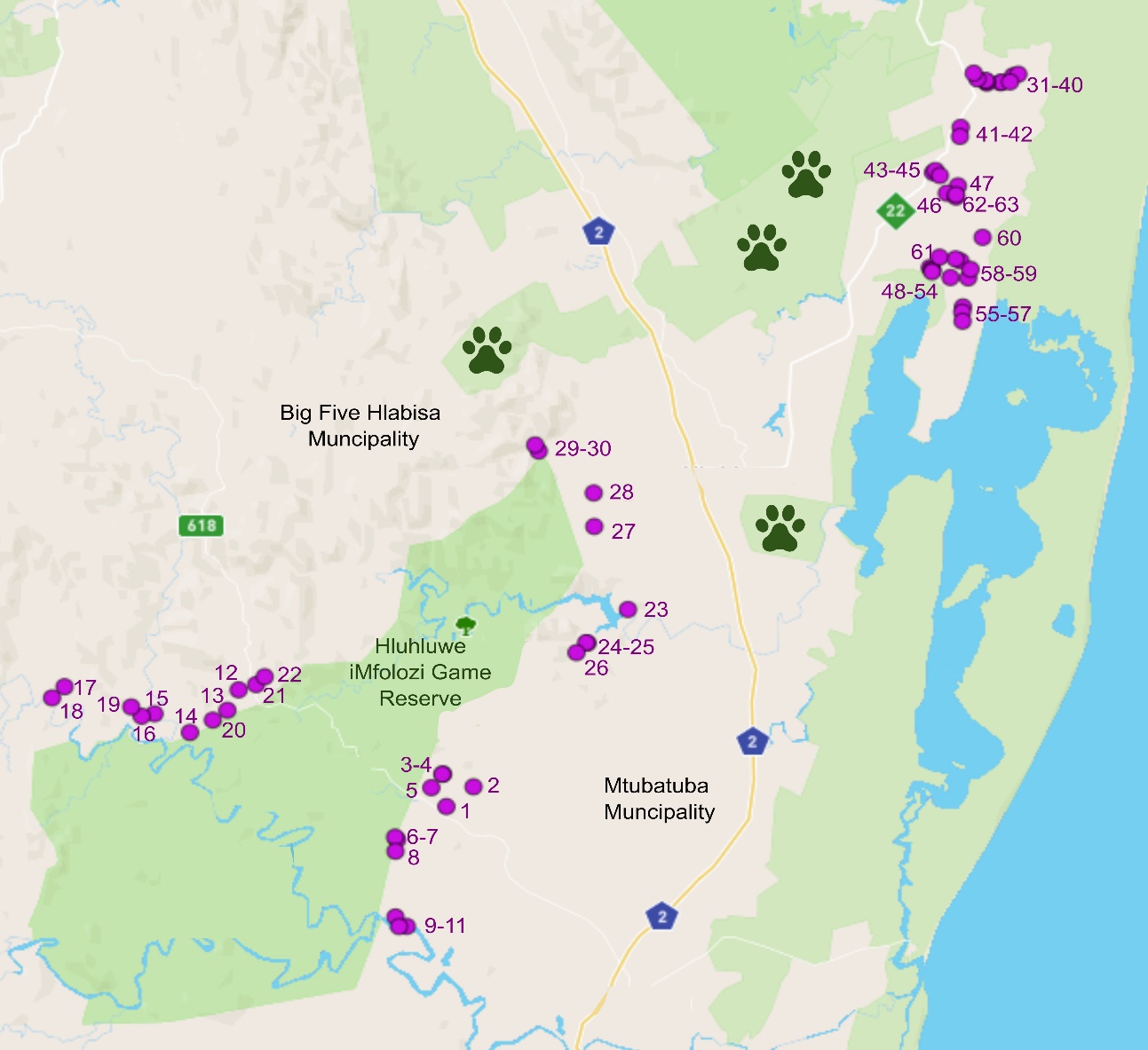
**

**Figure S2**. Map showing the locations of the 63 sampling sites in KwaZulu-Natal, South Africa, from which water margin samples were collected and screened for *Mycobacterium tuberculosis* complex (MTBC) DNA. Each site is marked with a circular location pin and the site number indicated. The locations of private game reserves are indicated with a paw print icon.
